# Significant linkage at chromosome 19q for otitis media with effusion and/or recurrent otitis media (COME/ROM)

**DOI:** 10.1186/1471-2350-12-124

**Published:** 2011-09-26

**Authors:** Wei-Min Chen, E Kaitlynn Allen, Josyf C Mychaleckyj, Fang Chen, Xuanlin Hou, Stephen S Rich, Kathleen A Daly, Michèle M Sale

**Affiliations:** 1 Center for Public Health Genomics, University of Virginia, Charlottesville, VA 22908, USA; 2 Department of Public Health Sciences, University of Virginia, Charlottesville, VA 22908, USA; 3 Department of Biology, University of Virginia, Charlottesville, VA 22908, USA; 4 Department of Medicine, University of Virginia, Charlottesville, VA 22908, USA; 5 Department of Biochemistry and Molecular Genetics, University of Virginia, Charlottesville, VA 22908, USA; 6 Department of Otolaryngology, University of Minnesota, Minneapolis MN 55455, USA

**Keywords:** Linkage, fine mapping, otolaryngology

## Abstract

**Background:**

In previous analyses, we identified a region of chromosome 19 as harboring a susceptibility locus for chronic otitis media with effusion and/or recurrent otitis media (COME/ROM). Our aim was to further localize the linkage signal and ultimately identify the causative variant or variants. We followed up our previous linkage scan with dense SNP genotyping across in a 5 Mb region. A total of 607 individuals from 139 families, including 159 affected sib pairs and 62 second-degree affected relative pairs, were genotyped at 1,091 SNPs. We carried out a nonparametric linkage analysis, modeling marker-to-marker linkage disequilibrium.

**Results:**

The maximum log of the odds (LOD) score increased to 3.75 (P = 1.6 × 10^-5^) at position 63.4 Mb, with a LOD-1 support interval between 61.6 Mb and 63.8 Mb, providing significant evidence of linkage between this region and COME/ROM. The support interval contains over 90 known genes, including several genes involved in the inflammasome protein complex, a key regulator of the innate immune response to harmful exogenous or endogenous stimuli. Parametric linkage analysis suggests that for a sib of an affected individual, the recurrence risk of COME/ROM due to this linkage region is twice the recurrence risk in the population. We examined potential associations between the SNPs genotyped in this region and COME/ROM, however none provided evidence for association.

**Conclusion:**

This study has refined the 19q region of linkage with COME/ROM, and association results suggest that the linkage signal may be due to rare variants.

## Background

Otitis media (OM), or inflammation of the middle ear, represents a leading reason for physician visits by children and a major component of the pediatric healthcare burden [1]. A significant proportion of children who suffer from acute OM go on to experience either chronic otitis media with effusion (COME) or recurrent otitis media (ROM). COME/ROM can result in permanent hearing loss, tympanic membrane abnormalities, other infections such as meningitis, parental days lost from work, and other sequelae [2,3].

Genetic factors play an important role in COME/ROM susceptibility. COME/ROM aggregates in families [4-7], and first-degree relatives of COME/ROM patients have higher rates of otitis media than would be expected based on population rates [8]. Twin and triplet studies confirm the strong familial component of COME and ROM, with heritability estimates of 0.64-0.74 in monozygotic twins and 0.20-0.53 in dizygotic twins [9-13].

To date, two linkage studies for COME/ROM have been published but the loci identified in the two studies did not overlap. The first genome-wide linkage scan, conducted by our group [14], identified evidence of linkage to chromosomal regions on 10q26 and 19q13, and support for linkage on 3p conditional on linkage at 10q and 19q. Casselbrant *et al*. identified linkage peaks on chromosome 17q12, 10q22, 7q33, 6p25, and 4p15 [15]. In order to localize the linkage signal on chromosome 19 and identify susceptibility genes for COME/ROM, we have conducted fine mapping across this region, and evaluated these SNPs for linkage and association.

## Methods

### Subjects

This study was conducted with Institutional Review Board approval at the University of Minnesota, Wake Forest University, and the University of Virginia, and adhered to the tenets of the Declaration of Helsinki. Subjects who had tympanostomy tube surgery for COME/ROM (probands) and their families were recruited for the study, which has been described previously [8,14,16]. An otolaryngologist performed an ear examination to determine presence of OM sequelae without knowledge of the subject's prior OM history. In addition, tympanometric testing was performed in subjects at three frequencies (226, 630 or 710, and 1400 Hz) to detect abnormal middle ear mechanics, and hearing was screened at 20dB for speech frequencies. The current analyses included all subjects from the initial linkage scan of Daly *et al*. ( [14], with the addition of six new families rascertained and recruited using the same criteria [14]. A total of 607 individuals from 139 families, including 159 affected sib pairs and 62 second-degree affected relative pairs, were used in analyses.

### SNP selection and genotyping

We selected 1,536 SNPs for genotyping, including 1,492 chromosome 19 SNPs chosen from a combination of tagging, nonsynonymous and synonymous coding SNPs, physical coverage, and putative copy number variation (CNV) interrogation. Forty-four Ancestry Informative Markers (AIMs) spaced across the genome were included to verify major ethnic group membership and to detect European stratification in the families. Genotyping was carried out by the Center for Inherited Disease Research (CIDR) using Illumina's GoldenGate assay [17].

### Statistical analyses

After removal of SNPs on the basis of poor genotype quality, missing data, excessive replicate and/or Mendelian errors [18-20], or monomorphic status, 1,091 SNPs were available for analyses. Deviations from Hardy Weinberg Equilibrium (HWE) in 223 unrelated individuals were determined using the exact test [18], and all SNPs were in Hardy-Weinberg equilibrium (P > 0.0001). Two monozygotic (MZ) twin pairs were detected and incorporated in the analysis. The computer program package Merlin [20] was used to identify and resolve inconsistencies within families.

The Kong and Cox linear nonparametric linkage (NPL) method [21] as implemented in Merlin was used for the NPL linkage analysis. Marker-marker linkage disequilibrium (LD) was modeled in the linkage analysis using the maximum-likelihood clustered marker approach [22] implemented in Merlin with thresholds of *r*^2 ^> 0.5 and *r*^2 ^> 0.2. We also explored other strategies to take into account the potential impact of LD on the validity of our linkage findings. We analyzed a subset of 90 families (out of 139 families in total) in which all affected individuals have complete parental data. This subset of data is expected to be free of the LD effect on linkage [22].

The Linkage and Association Modeling in Pedigrees (LAMP) method [23] was used for the parametric linkage analysis. In contrast to other parametric linkage methods, the LAMP method does not require the specification of the genetic model of the disease, and parameters such as the penetrance of each genotype (probability of affected status given genotype) and the frequency of the disease susceptibility allele can be estimated from the data.

In order to investigate whether the support for linkage on chromosome 19q can be explained by a common genetic variant in the region, we applied three family-based association tests, the Transmission/Disequilibrium Test (TDT) [24], the Generalized Disequilibrium Test (GDT) [25], and the more powerful Quasi-Likelihood Score test M_QLS _[26]. The TDT method examines the allele transmission disparity from heterozygote parents to their affected offspring. Although the TDT is viewed as the standard of family-based association tests, the presence of linkage without allelic association can result in an inflated false positive rate [27]. The GDT method generalizes the comparison between parent and offspring to all discordant relative pairs, including those families with incomplete parental data that cannot be handled in the standard TDT method. The GDT method also allows the adjustment of linkage in the association analysis. We included sex as a covariate and incorporated the identical-by-descent (IBD) statistics in the GDT analysis. The M_QLS _method also incorporates association evidence across families and has been shown to be more powerful in many settings than standard methods. The prevalence of otitis media is specified at 0.1 in M_QLS _and only 137 Caucasian families (the reported ethnicity has been verified with AIMs) are included in the M_QLS _analysis. We also imputed the HapMap [28] CEU SNPs to identify potential associated variants that were not included in our SNP panel [29].

## Results

**Figure 1:**
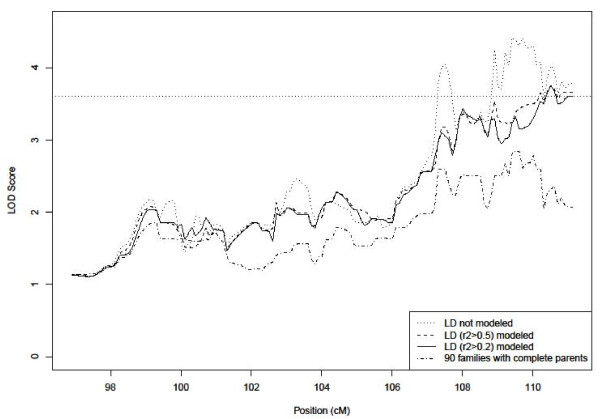
**LOD scores at 19q**. The dotted line indicates a non-parametric linkage scan without modeling marker-marker LD. The dashed and solid lines indicate non-parametric LOD scores with LD modeled at *r*^2 ^> 0.5 and *r*^2 ^> 0.2 respectively. The dot-dash line indicates the linkage scan when only a subset of 90 families (out of 139 families in total) with complete parental data were included in the analysis.

The maximum LOD score of the NPL linkage analysis was 3.75 (P = 1.6 × 10^-5^) at 110.5 cM (corresponding to 63.4 Mb in physical distance), when the marker-marker LD was modeled at either *r*^2 ^> 0.5 or *r*^2 ^> 0.2. This result supported significant evidence for linkage between the 19q region and COME/ROM (Figure 1). The LOD-1 support interval (the interval in which the LOD score is within 1 unit of its maximum, which is usually treated as a confidence region [30]) for the identified linkage was between 107.3 cM and 111.1 cM (61.6 Mb and 63.8 Mb in physical distance). When the marker-marker LD was not modeled, the maximum LOD score was 4.41 at 109.4 cM. The large difference of the maximum LOD scores between the two models (with LD modeled vs. not modeled) reflects the inflation of linkage due to the unaccounted marker-marker LD among the dense SNPs [22].

When 90 families (65% of total number of families) with complete parental data were included in the linkage analysis, the maximum LOD score was 2.85. Note the other 35% of data contains additional linkage evidence that can be estimated as shown above [22]. Since the marker-marker LD does not influence the linkage analysis in 65% of the data even without modeling LD in the analysis [22], and our result provides a projected LOD score of 4.38 for the entire set of families for the hypothetical case when all 138 families have complete parental information, the identified linkage in the 19q region is further supported.

The maximum LOD score based upon parametric linkage analysis was 5.42 at 107.5 cM, corresponding to P = 1.6 × 10^-5^. For a sibling of an affected individual, the recurrence risk of COME/ROM attributable to the 19q locus is twice as high as the recurrence risk in the population.

**Figure 2:**
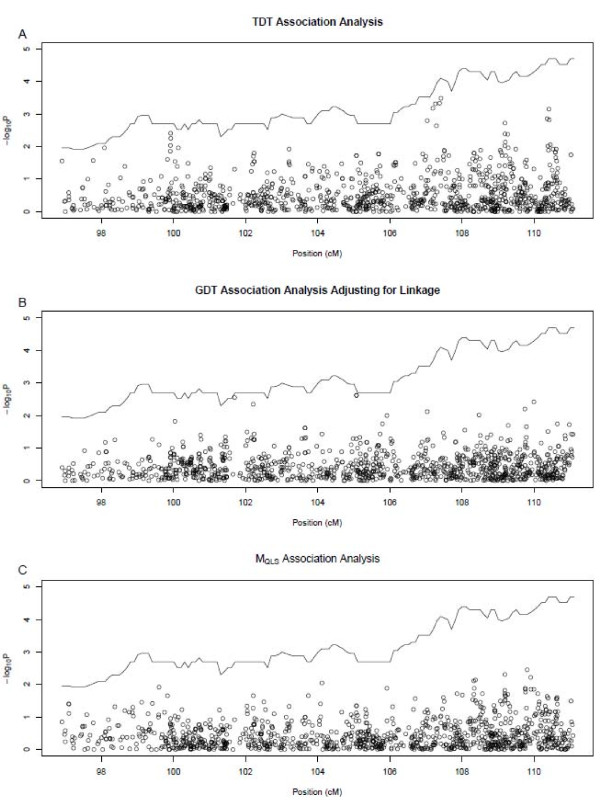
**P values of association from two association tests: A**. TDT, B. GDT, C. M_QLS_. P values in -log_10 _scale are represented by circles. The solid line indicates p values of non-parametric LOD scores with LD modeled at *r*^2 ^> 0.2.

No statistically significant (P < 0.0003) association between any SNP and COME/ROM was identified (Figure 2). Using a Bonferroni correction, 1,091 SNPs requires a nominal significance level of P < 4.6 × 10^-5^. Since the number of independent SNPs should be less than 1,091 because of the LD between SNPs, we estimated the number of independent SNPs by pruning pairwise LD between SNPs. There are 216 SNPs that are in approximate linkage disequilibrium with each other (r^2 ^< 0.1), and a Bonferroni correction based on 216 SNPs gives a nominal significance level of P < 0.00023. None of the observed associations reach either level of significance.

The five strongest associations identified through the TDT family-based association analyses (P value < 0.001) all fall in the LOD-1 support interval. In contrast, none of the family-based GDT or M_QLS _analyses yielded significant evidence of association (P < 0.001). The strongest association detected by TDT reflects the inflation of the statistics due to linkage in the region, rather than by association at a single variant [25]. Using GDT analysis, the strongest association (P = 0.0024) falls outside of the LOD-1 support interval.

Rare variants in this region of 19q are much less represented than common variants in our SNP panel. Among all 1,091 SNPs, there are only 81 SNPs with a minor allele frequency less than 5%. The lack of significant association is consistent with evidence that linkage in the 19q region is not due to common genetic variants.

## Discussion

We extended our previously reported linkage analysis on chromosome 19q with dense SNP genotyping in the critical 5 Mb region. Analyses of these additional data confirmed significant evidence supporting linkage in this region to COME/ROM, increasing the LOD score from 2.61 [14] to 3.75 at position 63.4 Mb. The SNP closest to D19S254 (62.359 Mb), the marker with the maximum LOD score of 2.61 in the original linkage scan [14], is rs810859 (62.358 Mb), and the LOD score at this SNP is 3.38 (with LD r^2 ^> 0.2 modeled). The dense markers, as well as a larger sample size, have therefore helped improve the LOD score and localize the region of linkage. Additionally, parametric linkage analysis of our data suggests that for a sibling of an affected individual, the recurrence risk of COME/ROM that is due to this linkage region is twice as high as the recurrence risk in the population. Our analysis provides evidence that a COME/ROM susceptibility locus can be found within this region.

The LOD-1 support interval represents the telomeric region of chromosome 19. This region is high in gene content, containing over 90 known genes. Many of the genes in this region are zinc finger and zinc finger-related genes. Potential candidates for COME/ROM susceptibility include *ZNF71*, an endothelial zinc finger gene induced by TNF-α [31], *ZNF8*, which represses BMP and FGFß pathways important during development {Jiao, 2002 #32}, and *ZNF304*, which has been found to activate lymphocytes [32]. Other candidate genes in this region include members of the inflammasome protein complex, *NLRP13, NLRP5 *and *NLRP8*. Inflammasomes are key regulators of the innate immune response to harmful exogenous or endogenous stimuli [33,34] Alpha-1-B glycoprotein (A1BG) is similar in sequence to proteins in the immunoglobulin supergene family, and has been associated with severe inflammation [35]. Chromatin modifying protein 2A (*CHMP2A*) is part of the chromatin-modifying protein/charged multivesicular body protein (CHMP) involved in surface receptor degradation and formation of endocytic bodies [36]. Clearly, there are many genes within the linkage peak region on chromosome 19 with biological functions of potential relevance to COME/ROM susceptibility. The actions of multiple genes in this region, rather than a single gene, on COME/ROM risk cannot be excluded.

## Conclusion

In summary, we confirmed linkage of COME/ROM to chromosome 19q in a family based population with dense genotyping of a previously identified 5 Mb region. The lack of significant association with common variants in this region suggests that the observed significant linkage may be due to rare variants. Numerous studies have provided evidence that rare variant are involved in the etiology of complex traits (Liu and Leal, 2010). Further examination of the 19q region in COME/ROM susceptibility by next-generation sequencing of the region may be required in order to detect rare variants that may have novel and functionally significant effects. These studies are currently underway to determine the COME/ROM susceptibility gene in this region.

## Authors' contributions

WMC, MMS, KAD and EKA drafted the manuscript. SSR and KAD conceived of the family-based study, and KAD oversaw recruitment. MMS, JCM, SSR, and KAD participated in the design of the linkage follow-up study. JCM was responsible for SNP selection. MMS oversaw and coordinated molecular genetic analyses. JCM and XH conducted quality control procedures on genotype data. WMC and FC performed the statistical analysis. All authors read and approved the final manuscript.

## Pre-publication history

The pre-publication history for this paper can be accessed here:

http://www.biomedcentral.com/1471-2350/12/124/prepub
